# Towards Active Tracking of Beating Heart Motion in the Presence of Arrhythmia for Robotic Assisted Beating Heart Surgery

**DOI:** 10.1371/journal.pone.0102877

**Published:** 2014-07-21

**Authors:** E. Erdem Tuna, Jamshid H. Karimov, Taoming Liu, Özkan Bebek, Kiyotaka Fukamachi, M. Cenk Çavuşoğlu

**Affiliations:** 1 Department of Electrical Engineering and Computer Science, Case Western Reserve University, Cleveland, Ohio, United States of America; 2 Department of Biomedical Engineering, Lerner Research Institute, Cleveland Clinic, Cleveland, Ohio, United States of America; 3 Department of Mechanical Engineering, Özyeğin University, Istanbul, Turkey; University of Minnesota, United States of America

## Abstract

In robotic assisted beating heart surgery, the control architecture for heart motion tracking has stringent requirements in terms of bandwidth of the motion that needs to be tracked. In order to achieve sufficient tracking accuracy, feed-forward control algorithms, which rely on estimations of upcoming heart motion, have been proposed in the literature. However, performance of these feed-forward motion control algorithms under heart rhythm variations is an important concern. In their past work, the authors have demonstrated the effectiveness of a receding horizon model predictive control-based algorithm, which used generalized adaptive predictors, under constant and slowly varying heart rate conditions. This paper extends these studies to the case when the heart motion statistics change abruptly and significantly, such as during arrhythmias. A feasibility study is carried out to assess the motion tracking capabilities of the adaptive algorithms in the occurrence of arrhythmia during beating heart surgery. Specifically, the tracking performance of the algorithms is evaluated on prerecorded motion data, which is collected *in vivo* and includes heart rhythm irregularities. The algorithms are tested using both simulations and bench experiments on a three degree-of-freedom robotic test bed. They are also compared with a position-plus-derivative controller as well as a receding horizon model predictive controller that employs an extended Kalman filter algorithm for predicting future heart motion.

## Introduction

The present work is focused on a previously unexplored aspect of robotic-assisted beating heart surgery; namely, evaluating performance of robotic active relative motion cancellation (ARMC) control algorithms for heart motion tracking under atrial fibrillation (AF) induced arrhythmia conditions. The occurrence of arrhythmia during cardiac procedures cannot be ruled out, hence robots must be able to handle this case to be clinically useful. This paper is the first to acknowledge this essential problem of tracking heart motion in the presence of heart rhythm irregularities during arrhythmia.

There is substantial amount of literature in heart motion tracking studies for robotic-assisted beating heart surgery [1]–[14] (see *Background* for further details). However, the studies under slow heart rate variations are limited [4], [14] and none of the heart motion tracking algorithms are evaluated under arrhythmia conditions.

The purpose of this study is to evaluate the feasibility of the ARMC control algorithms introduced previously in [11]–[14] under arrhythmia conditions. Specifically, a novel set of *in-vivo* heart motion data is acquired from a bovine model, where the data includes artificially induced atrial fibrillation rhythm irregularities. Then, the ARMC control algorithms are tested on this data using simulations and bench-top experiments on a hardware test-bed. The algotihms are further compared with an extended Kalman filter (EKF) based control algorithm and a position-plus-derivative (PD) control algorithm. Although the control algorithms are formerly described in [11]–[14] and the proposed approach for inducing arrhythmia might be a limited model for the clinical situation, the presented results provide novel contribution to the literature of heart motion tracking for robotic assisted beating heart surgery.

### Background

Coronary artery bypass graft (CABG) surgery requires surgeons to operate on blood vessels that move with high bandwidth. This rapid motion of heart makes it difficult to track these arteries by hand effectively [15]. Contemporary techniques either stop the heart and use a cardio-pulmonary bypass machine, *on-pump*, or passively restrain the beating heart with mechanical stabilizers, *off-pump*, in order to cancel the biological motion of the heart during CABG surgery. However, using on-pump CABG surgery might cause significant complications that might occur during or after surgery, which includes long-term cognitive loss [16]. Off-pump CABG surgery is mostly limited to the front surface of the heart and significant residual motion is observed during stabilization [17].

Robotic-assisted beating heart surgery has been proposed to enable high dexterity surgical manipulations to be performed on a beating heart without mechanical stabilization. In robotic-assisted beating heart surgery conventional surgical tools are replaced with robotic instruments, which are directly controlled by the surgeon through teleoperation. In this system, the surgeon views the surgical site through a camera mounted on a robotic arm, which follows the heart motion, giving a stabilized view. The robotic surgical instruments also track the heart motion, canceling the relative motion between the surgical site and the instruments. As a result, the surgeon operates on the heart as if it was motionless, while the robotic system actively compensates the relative motion of the heart [18].

Due to the high bandwidth of the heart motion, it is necessary to employ model predictive feed-forward control algorithms, which rely on estimation of the future motion of the point-of-interest (POI) to achieve sufficient tracking accuracy [1], [4], [11]. However, the performance of these algorithms under changes in heart rhythm is a valid and important concern.

Several approaches have been proposed in the literature for modeling and predicting the motion of a POI on the heart, including algorithms using harmonics models [1], extended Kalman filters [2]–[4], coupled breathing and heartbeat motion models [5], [6], surface deformation models [7]–[9], ECG synchronized periodic models [10], [11], and generalized adaptive filters [12]–[14]. However, heart motion tracking performance using these algorithms under heart rhythm irregularities has not been addressed in the literature.

In our previous work two least-squares-based prediction algorithms, namely one-step and generalized, using an adaptive filter to generate future position estimates, were introduced [14]. These algorithms were studied by using a range of prerecorded *in vivo* constant and slowly varying heart rate motion data. Our previous report showed that the tracking of POI motion is no longer the bottleneck since the necessary amount of root-mean-square (RMS) tracking error on the order of 100–250* µm* is achieved. Furthermore, if the heart behavior slowly changes, then adaptive predictors are able to adjust to these changes quickly enough and yield good tracking results.

This paper extends our previous efforts on active tracking of the beating heart to the case of arrhythmia presence, where heart behavior changes significantly and abruptly. Here, a feasibility study is performed to determine motion tracking capabilities of these algorithms in the occurrence of arrhythmia. We evaluate the tracking performance of one-step and generalized predictors presented in [14] used in conjunction with the receding horizon model predictive control algorithm (RHMPC) presented in [11] with a range of prerecorded *in vivo* arrhythmia data by simulations and on a three degree-of-freedom (3-DOF) robotic test bed. Additionally, tracking results of RHMPC with an EKF (adapted from the implementation presented in [4]) and of a PD controller are given for comparison purposes.

The rest of this paper is organized as follows. First, experimental setup and analysis of the arrhythmia data is described. Then, the adaptive prediction methods are briefly revisited, and the results and the comparison of the algorithms are presented. Finally, conclusions and possible extensions are given. The adaptive prediction algorithms are proposed in [14]; the applications of these algorithms to arrhythmia tracking is the original contribution and has not been published previously.

## Materials and Methods

### Analysis of Heart Data

The heart motion data were collected *in vivo* from three calves using a sonomicrometer and all of the bench-top experiments were performed with these prerecorded data. The size of the hearts were approximately 16
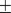
2 cm in length and 12
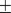
1 cm in width. In the experimental set-up for measurement of heart motion, two sonomicrometry crystals were placed on the epicardial surface. One crystal was sutured to the left side of the left anterior descending artery (LAD), whose location is referred to as “Anterior” in the rest of the paper. The second crystal was sutured on the right side of the LAD, whose location is referred as “Lateral” in the paper. Eight other crystals were asymmetrically mounted upon a rigid plastic base of diameter 60 mm, on a circle of diameter 50 mm, forming a reference coordinate frame. This rigid plastic sensor base was placed in a rubber latex balloon, which was filled with a 9.5% glycerine solution. The reason of using such a set-up was to ensure a continuous line of sight between the base crystals and the crystal on the heart surface through a liquid medium for proper operation of the sonomicrometry sensor system.

Two pacemaker electrodes were sutured to the right atrium and connected to a pacemaker to set a desired cardiac rhythm and induce arrhythmias. The method of inducing atrial fibrillation (AF) by electrically stimulating right atrium has been used in the literature for easy and reliable modeling of AF [19]–[21]. The AF was induced by stimulating the right atrium via two electrodes connected to an Electric Transformer with 2.5V AC @60 Hz. The ECG pattern of the AF (i.e. the absence of P waves, disorganized electrical activity in their place, and irregular R-R intervals due to irregular conduction iimparted to the ventricles) induced intraoperatively in our experimental setting was similar to that found clinically in patients [19]–[21]. This set-up for arrhythmia generation was chosen for motion control and tracking purposes only and not for the functional aspect of rhythm disturbances. The formulation of the biochemical and the pathophysiological aspects of the cardiac muscle motion, and the related neurostimulation are beyond the scope of the present study.

**Figure 1:**
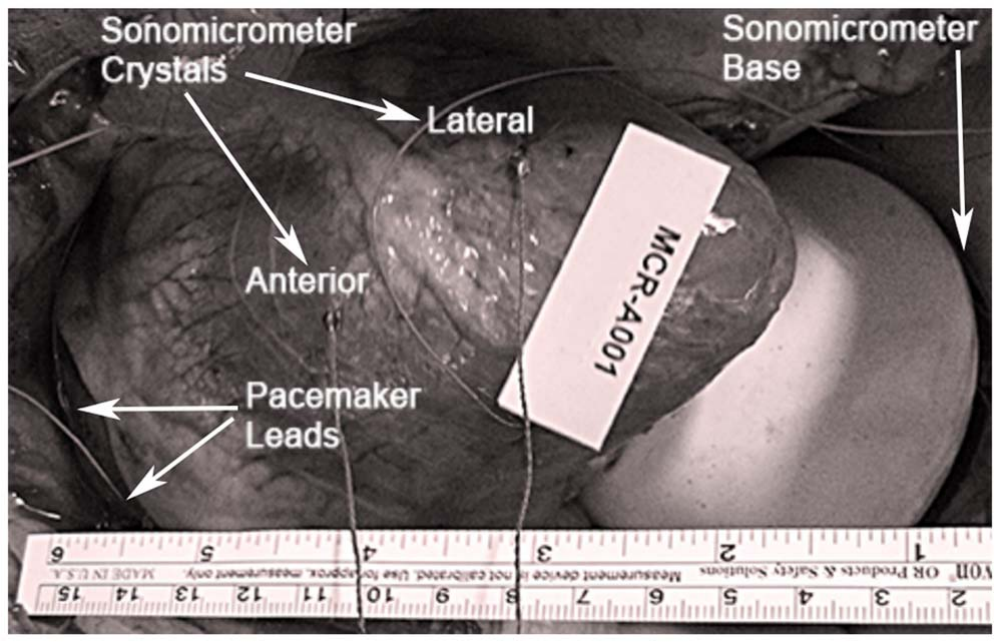
Experimental setup for the measurement of the heart motion. Two sonomicrometer crystals that are sutured on the anterior and posterior surfaces of the heart are used for data collection. Pacemaker leads and sonomicrometer base are also visible in the image.

**Figure 2:**
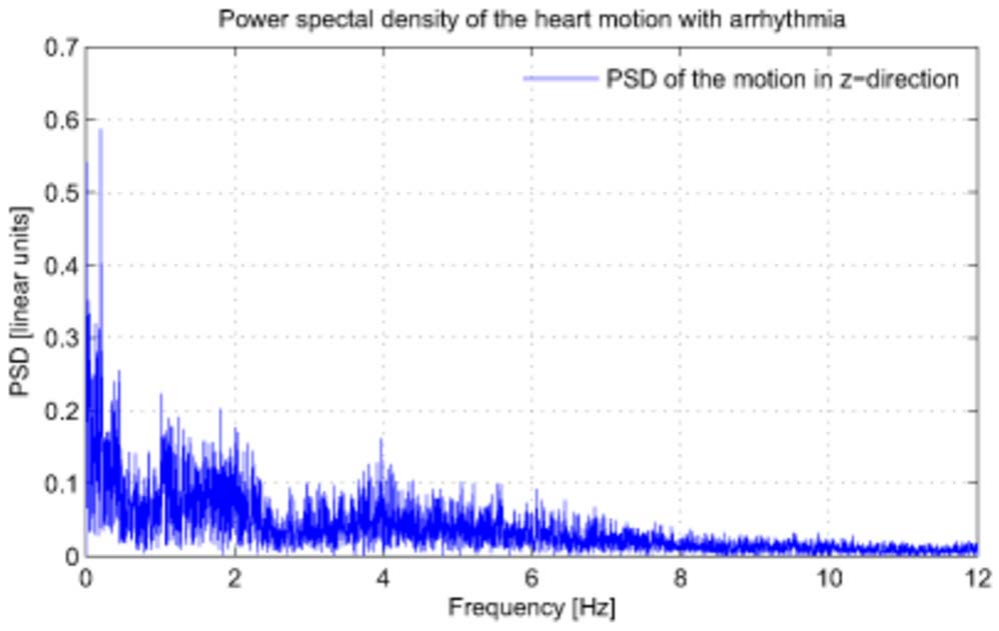
Power spectral density of the heart motion in the z-direction. Heart motion modes are inseparable. The frequency axis is set to 12-s arrhythmia data from animal 1 is shown. The spectrum corresponds to motion of the POI located at 0.5 cm on the right side of LAD.

Data were processed offline and only filtering performed on the data (using the proprietary software provided with the system) was very limited removal of the outliers, which occasionally occur as a result of ultrasound echoing effects. Description of the sonomicrometer crystal locations on heart with respect to the left anterior descending artery (LAD) are given in Table 1. Fig. 1 shows the experimental setup for data collection. The sonomicrometer crystals, the sonomicrometer base, and the pacemaker leads are visible. A Fourier transform (Fig. 2) of the 183 s heart signal data from animal 1 reveals the inherently non-periodic nature of heart motion during arrhythmia. The abundance of intermittent frequencies in the power spectral density (PSD) with the absence of tall, narrow peaks indicates this point.

**Table 1:** Arrhythmia Data.

DataSet (Body weight)	Animal 1 (75.8 kg)		Animal 2 (68.7 kg)		Animal 3 (70.2 kg)	
**Location of crystal on cardiac surface**	Anterior	Lateral	Anterior	Lateral	Anterior	Lateral
**Position relative to LAD**	2 cm left	0.5 cm right	1 cm left	5 cm right	1 cm left	5 cm right

Data from animal 1 have a sampling rate of 180 Hz with durations 85 s and 183 s respectively for “Anterior” and “Lateral” locations. Data from animal 2 have a sampling rate of 404 Hz with a duration of 85 s for both locations. Data from animal 3 have a sampling rate of 404 Hz with durations respectively 215 s and 105 s for “Anterior” and “Lateral” locations.

### Ethics Statement

The *in vivo* study protocol was approved by the Cleveland Clinics Institutional Animal Care and Use Committee, and all animals received humane care in compliance with the Guide for the Care and Use of Laboratory Animals (Institute of Laboratory Animal Resources, Commission on Life Sciences, National Research Council, National Academy Press, Washington, DC, 2011) and institutional guidelines.

### Adaptive Motion Estimation Algorithms

The most important aspects of robotic surgical instruments in robotic assisted CABG surgery are accurately measuring and predicting the heart motion as they are instrumental in tracking and canceling the relative motion between the heart surface and surgical tools attached to the robotic manipulators. The rapid motion of the heartbeat component possesses demanding requirements on the control architecture of the robotic system in terms of the bandwidth of the motion that needs to be tracked. Feed-forward control algorithms, which rely on estimations of upcoming heart motion, need to be utilized to achieve sufficient tracking accuracy [1], [4], [11].

**Figure 3:**
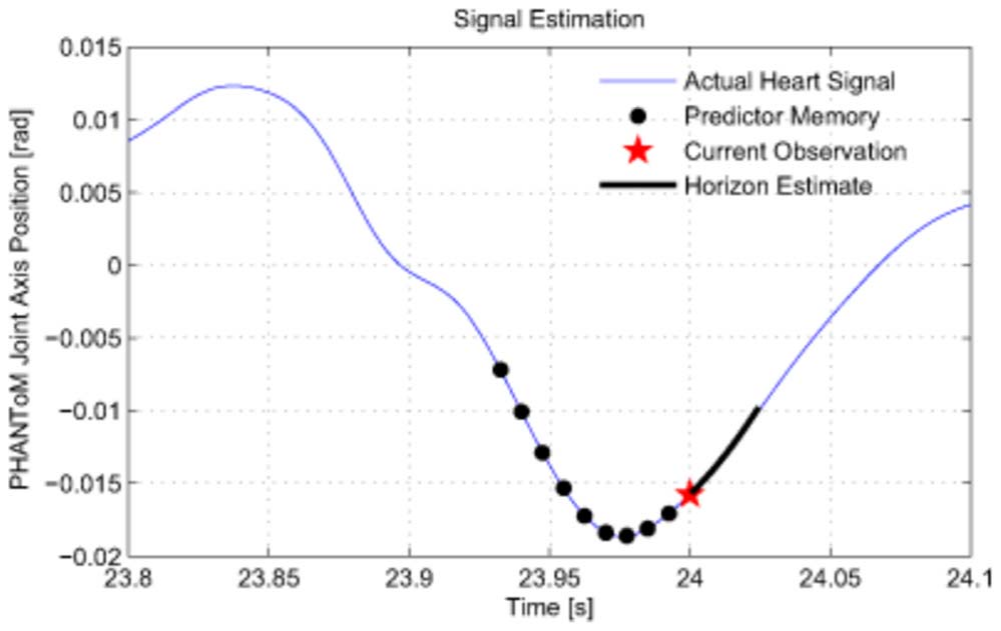
A schematic of the heart motion prediction problem. The circles represent past observations, now in memory, the 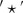 is the current observation, and the short curve originating from there is the horizon estimate. The predictor takes the past observations and produces the horizon estimate from past observations.

Two adaptive filter-based predictors used in this study provide an estimate of the immediate future of the POI motion over a prediction horizon to the feed-forward control algorithm. The best estimate is defined to be the one that minimizes the square of the estimation error, where the estimation error is the difference between the prediction and the observed value at that time. Once a method is established to predict the next observations, a sequence of future observations can be estimated. Fig. 3 provides a graphical schematic of the prediction problem.

The implementations of the predictors parameterize a linear system to predict POI motion and rely on recursive least-squares (RLS) adaptive filter algorithms. The one-step predictor assumes a linear system relation between the consecutive samples in the prediction horizon, whereas the generalized method performs the parametrization of the linear system independently for each point throughout the horizon.

The estimated trajectory of the POI on the heart is used as a feed-forward control signal in a model predictive controller [22]–[25]. The RHMPC algorithm combines linear quadratic optimal control strategy with prediction. The robot model and the estimated trajectory of the POI, which extends into the future, provide the prediction. At each time step, the control action is calculated by solving a finite horizon linear quadratic optimal control problem, which compares the predicted plant signals with the provided desired trajectory and the control objectives for the given time horizon.

The details of the RHMPC algorithm can be found in [11]. The motion estimation problem and adaptive estimation algorithms are comprehensively explained in [14]. They have been omitted here due to space constraints.

In the authors' previous work [14] the effectiveness of the RHMPC algorithm, which employs adaptive predictors to estimate upcoming heart motion, was studied during regular and slowly-changing heart rate conditions. Building on this work, this study focuses on the problem of heart motion tracking in the presence of highly irregular heart rhythm conditions. Specifically, the tracking performance of the algorithms is evaluated with arrhythmia data. To the best of the authors' knowledge, there are no previous studies in the literature to address this problem.

## Results

It is necessary to evaluate algorithms on some type of hardware testbeds. The algorithms were tested on a PHANToM Premium 1.5A haptic device, which is a 3-DOF robotic system. The PHANToM provides insight into the effectiveness of the algorithms on a desired system. Its lightweight frame and drive system allow for sufficient motion and speed to attempt to track the heartbeat signal. With its high bandwidth and low inertia features, the PHANToM robot possesses characteristics similar to those desired in a surgical robot that would be capable of heart motion compensation. Such a surgical robot would need to have lightweight links, low inertia design, and low-friction actuation system for tracking the heart with sufficient motion and speed. Such a prototype is currently being developed [26].

In the experimental setup, control algorithms were executed in xPC Target and run in a real-time kernel with a sampling time of 0.5 ms on a Intel Xeon 2.33 GHz Core PC. The nonlinearities of the test-bed system (i.e., gravitational effects, joint frictions, and Coriolis and centrifugal forces) were canceled independently from the controller (more details can be found in [27]). The robot was commanded to follow the combined motion of heartbeat and breathing. The system used online streaming position data in place of real-time measurements.

The robot model was controlled using RHMPC. The encoder positions on the PHANToM were recorded and these positions were transformed into end effector positions. The reported RMS errors, which represent the tracking performance of the algorithms, were calculated from the difference between the prerecorded target point and the actual end effector position calculated from joint angles.

In both simulations and experiments, the same control methods and reference data were used. During the trials, a 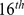 order correlated signal one-step estimator and a 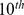 order generalized estimator were used. The predictors were downsampled by a factor of 15, processing observations that were 7.5 ms apart [14] and used to predict a 25 ms horizon. The 25 ms horizon corresponds to 50 control samples into the future. The length of the control horizon and estimator orders were chosen for the optimum error/performance under real-time computation requirements [11], [14].

As sonomicrometer was employed to acquire heart motion data used in this study, the sensor measurement delays were not considered, while determining the 25 ms prediction horizon. The sonomicrtometry sensor operates at high update rates (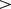
125 Hz) and has a high spatial resolution (24*µm*) [28]. The primary sources of measurement error include sonomicrometry crystal geometry, ultrasound echoes, and either weak or missing signals [29]. In this sense, sonomicrometry system architecture does not cause significant data acquisition and processing delays, unlike the vision-based and the ultrasound-based sensory systems, where there are inherent to image acquisition and processing delays [14], [30]. When such image-based sensor modalities are employed as in [4], the non-negligible delays introduced due to image acquisition and processing must be compensated. Thus, 25 ms estimation horizon is almost exclusively used for feed-forward compensation of the robot dynamics for improved control performance in this study.

For each case, experiments on the PHANToM robot were run 10 times with the estimation algorithms and again with the actual heart motion data as future signal reference for the prediction horizon. The latter case represents a ‘perfect’ estimation, providing a performance base of the robotic system's capability. It was noted that the deviation between the trials had been very small. Among these results, the maximum values for the *End-effector RMS and Maximum Position Errors in millimeters* in 3D and *RMS Control Effort in millinewton meters* are summarized respectively in Table 2 and Table 3 for the simulations and respectively in Table 4 and Table 5 for experiments to project the worst cases. The *RMS magnitude* and *peak-to-peak amplitude* of the heart motion for each data set are also stated to highlight the importance of using motion prediction for decreasing the tracking error.

**Table 2:** Simulation Results for End-Effector Tracking: RMS End-Effector Error and MAX Position Error for the Control Algorithms.

End-effector Tracking Results	RMS Position Error [mm]					
	(Maximum Position Error [mm])					
DataSet	Animal 1		Animal 2		Animal 3	
Crystal Location	Anterior	Lateral	Anterior	Lateral	Anterior	Lateral
**P-P amp of POI motion [mm]**	11.811	20.946	7.619	10.399	13.796	18.676
**RMS mag of POI motion [mm]**	4.488	4.419	3.238	3.063	4.819	4.058
RHMPC with Exact Reference Information	0.201	0.283	0.188	0.224	0.392	0.310
	(1.066)	(1.689)	(1.132)	(1.062)	(1.934)	(4.479)
RHMPC with One-Step Adaptive Filter Estimation	0.204	0.300	0.193	0.239	0.388	0.334
	(1.603)	(2.431)	(1.325)	(1.554)	(1.970)	(6.223)
RHMPC with Generalized Adaptive Filter Estimation	**0.192**	**0.262**	**0.185**	**0.221**	**0.380**	**0.299**
	(1.402)	(1.751)	(1.054)	(1.012)	(1.792)	(3.671)

**Table 3:** Simulation Results for End-Effector Tracking: RMS Control Effort for the Control Algorithms.

End-effector Tracking Results	Control Effort [mNm]					
DataSet	Animal 1		Animal 2		Animal 3	
Crystal Location	Anterior	Lateral	Anterior	Lateral	Anterior	Lateral
RHMPC with Exact Reference Information	12.965	15.649	10.943	13.894	17.425	15.761
RHMPC with One-Step Adaptive Filter Estimation	19.827	34.248	16.723	22.823	33.500	27.034
RHMPC with Generalized Adaptive Filter Estimation	15.294	21.475	11.861	15.095	23.003	20.195

**Table 4:** Experimental Results for End-Effector Tracking: RMS End-Effector Error and MAX Position Error for the Control Algorithms.

End-effector Tracking Results	RMS Position Error [mm]					
	(Maximum Position Error [mm])					
DataSet	Animal 1		Animal 2		Animal 3	
Crystal Location	Anterior	Lateral	Anterior	Lateral	Anterior	Lateral
**P-P amp of POI motion [mm]**	11.811	20.946	7.619	10.399	13.796	18.676
**RMS mag of POI motion [mm]**	4.488	4.419	3.238	3.063	4.819	4.058
RHMPC with Exact Reference Information	0.260	0.301	0.274	0.248	0.303	0.309
	(0.809)	(1.933)	(1.412)	(0.779)	(1.276)	(9.191)
RHMPC with One-Step Adaptive Filter Estimation	0.293	0.403	0.278	0.263	0.405	0.388
	(1.459)	(5.911)	(1.643)	(1.513)	(3.383)	(8.289)
RHMPC with Generalized Adaptive Filter Estimation	**0.282**	**0.340**	**0.276**	**0.247**	**0.319**	**0.330**
	(1.038)	(2.703)	(1.405)	(1.174)	(1.442)	(8.620)
RHMPC with Extended Kalman Filter Estimation	0.570	0.839	0.546	0.561	1.179	0.913
	(5.033)	(6.702)	(2.630)	(3.342)	(6.499)	(12.430)
PD Controller	0.465	0.775	0.419	0.484	1.130	0.886
	(3.674)	(6.026)	(2.207)	(4.610)	(7.227)	(12.231)

**Table 5:** Experimental Results for End-Effector Tracking: RMS Control Effort for the Control Algorithms.

End-effector Tracking Results	Control Effort [mNm]					
DataSet	Animal 1		Animal 2		Animal 3	
Crystal Location	Anterior	Lateral	Anterior	Lateral	Anterior	Lateral
RHMPC with Exact Reference Information	29.284	38.286	28.734	32.217	48.327	43.298
RHMPC with One-Step Adaptive Filter Estimation	34.416	51.048	29.813	31.574	54.779	43.241
RHMPC with Generalized Adaptive Filter Estimation	32.289	43.109	29.358	35.092	50.758	45.348
RHMPC with Extended Kalman Filter Estimation	35.092	48.341	32.947	32.657	67.176	55.756
PD Controller	49.938	83.809	42.894	57.258	140.301	99.273

**Figure 4:**
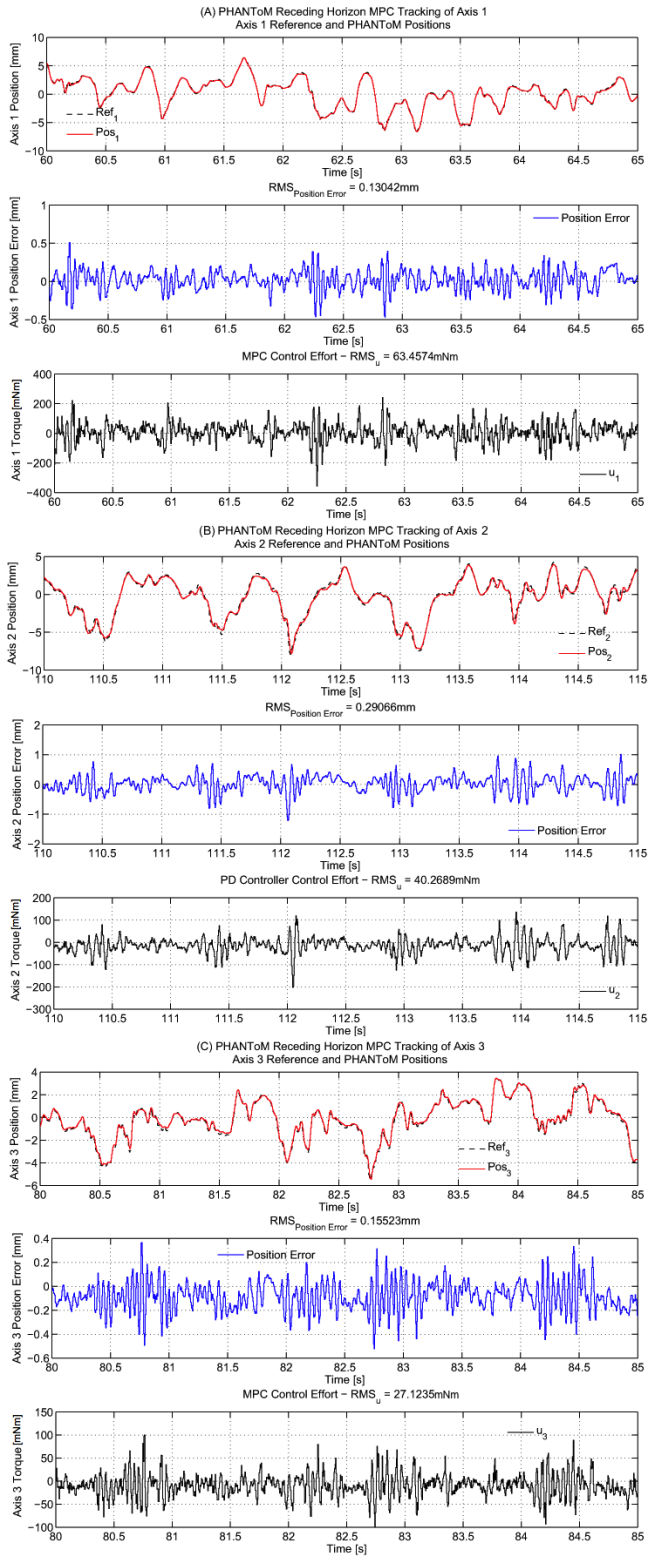
Tracking results of 183-s arrhythmia data (only a part of the data is presented) from animal 1 for the generalized predictor. Reference and PHANToM positions, RMS position error and MPC control effort are shown (A) Axis 1 results. (B) Axis 2 results. (C) Axis 3 results.

**Figure 5:**
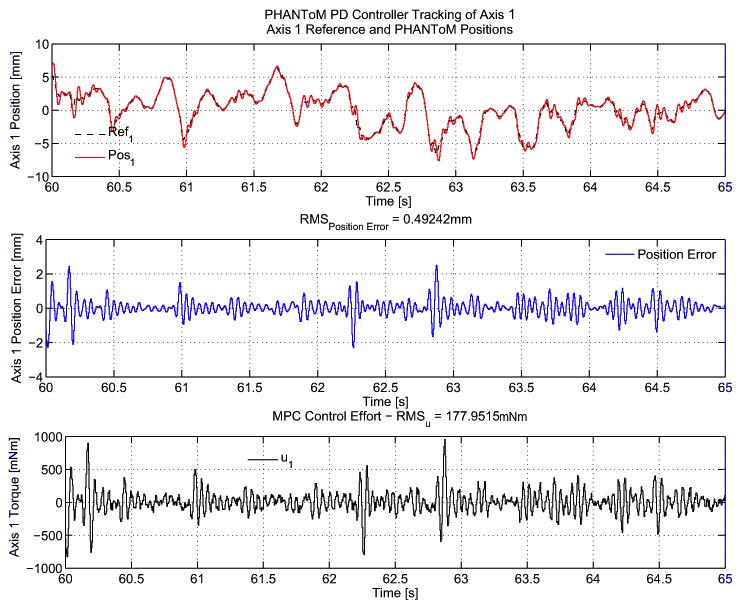
Tracking results of 183-s arrhythmia data (only a part of the data is presented) from animal 1 for the PD Controller. Axis 1 results are shown only.

Tracking results of 183-s arrhythmia data from animal 1 for the generalized predictor are shown in Fig. 4 for each PHANToM axis. Experimental results of the RHMPC with EKF predictor and PD controller are also given in Table 4 and Table 5 for comparison purposes (EKF predictor was implemented as described in [4] with the experimental parameters presented in [14]. More details on the PD controller can be found in [31]. Implementation details are omitted here due to space constraints). Tracking result for Axis 1 of PD controller is shown in Fig. 5.

The weighting parameters of the optimal index in RHMPC algorithms and the position and derivative constants in PD control were tuned to minimize RMS tracking error in both simulations and experiments. The tuning was performed individually for simulations and experiments. In experiments, additional tuning was performed to avoid the high frequency resonances so that no high frequency vibrations would be reflected to the structure.

In simulations, the generalized adaptive estimator gave better results than the exact heart signal in terms of RMS end-effector error (Table 2). This is likely due to the combination of two factors. First, the simulation model is a linearized and reduced order model of the actual hardware, hence does not completely reflect the influence of the nonlinearities of the hardware on the tracking performance. Second, the adaptive estimator has a robustness characteristic that makes its output (i.e. predicted trajectory) less susceptible to the noise existent in the raw motion data [12]–[14]. Accordingly, RHMPC with generalized estimator yields better results in the linear case. However, when the experiment is performed on the hardware, the effects of the nonlinearities have become apparent and the performance of the estimator-driven controller decreases (Table 4). It should be noted that although the simulation provides valuable insight about the effectiveness of the controller, the experimental trials are the best indicator of performance.

The experimental results reveal that adaptive predictors provide satisfactory tracking performance for the arrhythmia trajectories considered (Table 4). When the tracking results of the adaptive predictors are compared with each other, the generalized predictor gave better results than the one-step predictor in all experiments and yields close results to the the controller with exact heart signal reference. The RHMPC with adaptive estimation algorithms also outperformed RHMPC with EKF algorithm and PD controller.

In [4], Yuen et al. used an EKF algorithm with a quasi-periodic motion model to predict the path of mitral valve motion in order to compensate the time delay resulting from the 3-D ultrasound (3DUS) measurements. They concluded that since the EKF explicitly models the quasi-periodic motion of the heart, it can adjust to normal variations in heart rhythm. In [32], they noted that this does not extend to cases of arrhythmia, in which the motion of the heart is inherently non-periodic. Tracking results of RHMPC with EKF algorithm in Table 4 agree with this remark.

It is important to note that, RHMPC heavily relies on the estimate of the immediate future of the POI motion and the tracking problem is reduced to predicting the estimated reference heartbeat signal, when RHMPC has high enough precision to perform the necessary tracking [11]. Since EKF yielded poor predictions of the future POI position due to the unpredictable nature of heart during arrhythmia, the RHMPC with EKF algorithm was not able to track heart motion accurately. As a result, the PD controller yielded better performance than RHMPC with EKF algorithm.

The means of the RMS position errors and the standard deviations for RHMPC with generalized predictor, RHMPC with EKF, and PD controller are respectively computed as: 0.299 mm
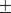
0.036 mm, 0.768 mm
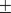
0.255 mm, 0.693 mm
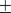
0.285 mm. These results further emphasize the better performance and robustness of RHMPC with generalized predictor on heart motion tracking during irregular beating. The effects of using RHMPC with generalized predictor on the arrhythmia tracking were also tested for statistical significance by paired t-test. For the results presented in Table 4, RHMPC with generalized predictor has led to improvements in tracking when compared to RHMPC with EKF (P
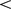
0.004) and PD controller (P
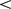
0.009).

We conjecture that the maximum error values are affected from the noise in the sensor data. In particular, the sonomicrometer measurements are susceptible to non-Gaussian noise resulting form echo effects and interruptions in ultrasound transmission paths that appear as large spikes or jump discontinuities. It is unlikely that the POI on the heart is capable of moving as much as 12 mm in a few milliseconds as it can occasionally be observed in the experimental data. More aggressive filtering can be performed to eliminate such high frequency motions (e.g. [33]–[35]), but the data has been kept as-is without applying any filtering to eliminate these jumps, as currently we do not have an independent set of sensor measurements (such as from a vision sensor) that would validate this conjecture. Though, it should be noted that such noise occurs only occasionally, as it can be observed through the significantly lower RMS error values, relative to maximum error values. At these high jumps, RHMPC algorithms with adaptive predictors outperformed PD controller, because with the PD controller these jumps initiated system oscillations (Fig. 5). Also, when the RMS control efforts are compared, the PD controller performed poorly (Table 5).

## Discussions

In this paper, heart motion tracking with adaptive estimation algorithms in the presence of arrhythmia is presented. Performance of the algorithms are evaluated with a range of data. These algorithms were previously explored under slowly varying heart rate conditions in [14]. To the best of our knowledge, no previous POI motion tracking work (see *Introduction*) has presented results in the presence of arrhythmia. The experimental RMS errors on the order of 0.250–0.340 mm obtained using the generalized estimator (in comparison to the RMS tracking errors on the order of 0.170–0.350 mm in constant heart rate and 0.160–0.180 mm in slowly varying heart rate conditions, as reported in [14]) represent satisfactory tracking performance during arrhythmia. Results show that if the behavior of the heart changes abruptly, the predictors are able to adapt the new heart behavior and can track the ideal time-varying solution. Although the resulting tracking errors are above the desired specification for effectively performing anastomosis, the system still achieved sufficient tracking accuracy to maintain safe tracking, until the system can potentially switch to a safe mode of operation. The results of the study should also be validated *in vivo*. An *in vivo* validation study would be valuable to verify that the proposed scheme will be effective under practical constraints of an operating room setting.

It is important to note that a certain level of consistency was necessary during the experimental setting of this study. For this reason, atrial fibrillation and its electrocardiographic pattern have been chosen as an arrhythmic scenario to provide a comparable and repeatable type of cardiac rhythm disturbance. The validity of the presented approach on other types of the peri-operative rhythm disturbance like tachycardia or sinus bradycardia will be explored in future studies. In addition, atrial fibrillation is a complex type of arrhythmia with high ventricular rates. Accordingly, the baseline heart rate (HR) is an important variable that was continuously monitored throughout the study. Yet, since the HR values may vary in each and every given setting, the high variability of the baseline heart rate was not considered to be a drawback of the experimental approach in choosing a bovine model. Similar to humans, the HR may vary per baseline and intra-operative hemodynamic conditions, but no interference and/or vagal tone dominance was observed during the studies. However, if the native cardiac pacing is altered, then the vagal tone and correlated effects may become more significant in heart failure model.

We also note that the presented study is not intended to evaluate the tracking performance of the algorithms on each and every type of atrio-ventricular arrhythmia. Rather, by simulating one particular type of arrhythmia from a wide variety of patterns, we aim to show feasibility of employing adaptive estimation algorithms for heart motion tracking in the occurrence of arrhythmia during beating heart surgery. The underlying electro-physiological mechanisms of arrhythmia are very complex. Atrio-ventricular contraction is affected by multitude of factors, such as, changes in the function of ion channels in atrial cells. The complex pattern of electrical impulse propagation within atrio-ventricular tissues is also determined by the frequency of stimulation and summation of waveforms. As such, constructing *in vivo* models of arbitrary arrhythmia types is not practical, if not impossible. A more broad *in vivo* study would overcome the limitations of the employed method of artificially inducing arrhythmias, and would cover more diverse situations in CABG surgeries and so will evaluate the performance and robustness of the proposed method in a wide range of conditions. Additionally, the applicability of the proposed system to specific arrhythmia patterns would be considered in further studies as a part of the development and safety control evaluation.

One way to improve tracking quality is to incorporate other types of data into the estimation scheme. One such possibility is to include the electrocardiogram (ECG) signal into the observations. In this way, the predictor is able to use the electrical signals that activate heart contraction in order to improve the prediction as in [11]. The ECG signal is very suitable for period-to-period synchronization with sufficient lead time for the feed-forward control, and identification of arrhythmias. If an arrhythmia is detected, feed-forward component of the controller can be turned off and switched to a further fail-safe mode if necessary, which in turn may improve performance during heart rhythm abnormalities.

Future works will also include multi sensor fusion where complementary and redundant sensors will be used for superior performance and safety, e.g., a vision based sensor system could be used as a secondary sensor for the *in vivo* validation of the proposed concept. Merging the sensor data from multiple position sources would increase accuracy of the motion estimation and improve tracking results. Adding more mechanical sensors that measure heart motion would improve the measurement precision and help to resolve calibration problems of the sonomicrometry system.
